# Impact of the use of local fidaxomicin treatment algorithms for managing *Clostridium difficile* infection in hospitalized patients in southeastern United States

**DOI:** 10.1186/s12941-018-0288-3

**Published:** 2018-10-11

**Authors:** Jonathan C. Cho, Sandy J. Estrada, Jamie J. Kisgen, Angelina Davis, Laura Puzniak

**Affiliations:** 1 College of Pharmacy, The University of Texas at Tyler, 3900 University Blvd., Tyler, TX 75799 USA; 2 Clinical Affairs, T2 Biosystems, Inc., Lexington, MA USA; 3 Department of Pharmacy, Sarasota Memorial Health Care System, Sarasota, FL USA; 4 Department of Pharmacy, WellStar Cobb Hospital, WellStar Health System, Austell, GA USA; 5 Merck & Co., Inc., Kenilworth, NJ USA

## Abstract

**Background:**

*Clostridium difficile*-associated diarrhea (CDAD) is a major public health threat that results in increased length of stay, hospital readmissions, deaths, and economic burden. CDAD treatment is often guided by severity of disease. Although various tools exist to determine CDAD severity, real-world data evaluating the use of such tools in treatment algorithms are sparse.

**Methods:**

A local CDAD treatment pathway was developed independently to guide fidaxomicin prescribing at wellStar Health System (WellStar) and at Lee Health (LH) and Sarasota Memorial Hospital (SMH). Each algorithm was designed locally by the stewardship pharmacist and was utilized to identify patients at high risk for *C. difficile* recurrence. Patient and clinical data was retrospectively gathered to evaluate the utility and outcomes of the treatment pathway.

**Results:**

There were 262 patients that received fidaxomicin at these three hospitals during the study time period. Only 30% at WellStar and 20% at LH or SMH met the study criteria and adhered to the pathway requirements. After completion of fidaxomicin, 30-day recurrence rates at WellStar was 0 and at LH and SMH 7%. Clinical cure rates were 83% in WellStar and 93% in LH and SMH.

**Conclusions:**

The results from these two pathways show positive outcomes for the use of fidaxomicin in patients at high risk for CDAD recurrence. This data supports the potential utility of fidaxomicin against CDAD.

## Background

### Burden of *Clostridium difficile* infection

Diarrheal illness and complications caused by *Clostridium difficile* associated diarrhea (CDAD) constitute a substantial and increasing burden to patients and healthcare facilities in the United States (US) and throughout the world. In the US, it has been estimated that there may be over 450,000 incident infections annually, with 83,000 of these being first recurrences, and over 29,000 cases leading to mortality [1]. Of these cases, approximately 100,000 cases are acquired in hospitals [2]. *C. difficile* imposes numerous burdens on resources including the need for private rooms, isolation supplies, enhanced environmental cleaning, and vigilant hand hygiene, resulting in an estimated excess cost of as much as $4.8 billion to US acute care facilities, in addition to severe negative impact on patients’ quality of life [3].

### Current CDAD therapies

Historically, treatments for CDAD included vancomycin and metronidazole, with the choice of therapy usually dependent on disease severity; tigecycline can also be used in cases of CDAD colitis [4]. Updated guidelines for the treatment of CDAD from the Infectious Diseases Society of America and Society for Healthcare Epidemiology of America were recently published and highlight the challenges in the prevention, diagnosis and treatment management of this condition. The guidelines recommend vancomycin and fidaxomicin over metronidazole for initial non-severe and severe episodes, initial recurrence and multiple recurrences and discusses the use of risk factors to identify patients that have increased likelihood for unfavorable outcomes [5].

Increasing incidence of treatment failure with traditional agents (i.e. metronidazole and vancomycin) prompted research into and development of new molecules. Fidaxomicin, an oral macrocyclic antimicrobial that inhibits *C. difficile* growth as well as sporulation through RNA transcription inhibition, and whose narrow spectrum minimizes alterations of the colonic microbiota compared to existing agents [6], was approved for treatment of non-severe CDAD in the US in 2011. Approval followed two large randomized controlled trials that showed fidaxomicin was non-inferior to vancomycin in terms of the rate of clinical cure, but it was associated with a lower recurrence rate at 6 weeks post-treatment [7, 8]. A recent Cochrane review of 22 studies encompassing 3215 patients found that 71% (407 of 572) of fidaxomicin patients achieved symptomatic cure, compared to 61% (361 of 592) of vancomycin patients (RR 1.17, 95% CI 1.04–1.31; moderate quality evidence) [9].

### CDAD treatment pathways

Despite its demonstrated efficacy and effectiveness, however, it has been difficult to precisely define fidaxomicin’s place in the CDAD armamentarium, due to unclear definitions of risk factors for recurrence or severe/complicated CDAD, lack of clinical data in multiple recurrences or complicated CDAD, and high economic cost [6]. For maximum benefit in terms of both patient outcomes and economic efficiency, it may be beneficial for institutions to develop and rigorously evaluate clinical pathways that can guide selection of fidaxomicin for those patients most likely to respond best to the drug, both in regard to acute symptom relief and prevention of recurrence. This is problematic, however, in that pathway centered efforts have met with only mixed success, due to problems such as prescriber noncompliance [10]. Achievement of adequate sample size so that reliable conclusions can be drawn can also present a difficulty in conducting such research. This paper presents an evaluation of the results of two fidaxomicin-directed treatment pathways implemented in two separate healthcare systems in the southeastern United States.

Various *C. difficile* disease scoring systems exist including a disease severity measure in the IDSA guidelines [11], the ATLAS system predicting cure [12], and the D’Agostino model for predicting recurrence [13]. The ATLAS scoring tool is a validated tool that takes into account the clinical factors of age, treatment with systemic antibiotics during CDAD therapy, leukocytosis, albumin, and serum creatinine at the time of diagnosis. This tool has been shown to be useful in predicting treatment response and mortality in CDAD and has potential to be a resource for evaluating severity and determining treatment selection [14]. The D’Agostino model is a simple scoring rule, developed to help predict the risk of developing CDAD recurrence based on the clinical features of age, number of unformed bowel movements, serum creatinine, episode of CDAD, and treatment choice [13]. Due to questionable reliability of documentation of unformed bowel movements, evaluation of the D’Agostino criteria, without assessing unformed bowel movements, as risk stratification measures is warranted. Each of these tools has the potential to help guide drug therapy for *C. difficile* infections; however, there is a lack of data evaluating the implementation of these tools in a real world setting. This study describes the local implementation and outcomes associated with two different fidaxomicin treatment pathways.

## Methods

A local CDAD treatment pathway was developed independently to guide fidaxomicin prescribing at WellStar Health System (WellStar) in Georgia and at Lee Health (LH) and Sarasota Memorial Hospital (SMH) in Florida. Each algorithm was designed locally by the stewardship pharmacist and was utilized to identify patients at high risk for *C. difficile* recurrence. Patient and clinical data was retrospectively gathered to evaluate the utility of the treatment pathway.

WellStar is a not-for-profit system of eleven hospitals serving NW metropolitan Atlanta. Patients with a positive *C. difficile* PCR result from clinical microbiology testing with Xpert *C. difficile*/Epi (Cepheid, Sunnyvale, CA) and a clinical diagnosis consistent with CDAD between 12/1/12 and 3/31/14 from 5 of the hospitals in the WellStar system were included in this study. Patients over age 18 were included if it was their initial episode or first recurrence of CDAD with laboratory confirmed, non NAP1 strains with less than 24 h of enteral (PO) vancomycin or parenteral (IV) or PO metronidazole and at least one of the following conditions: age > 65 with severe disease, ulcerative colitis/Crohn’s disease, creatinine clearance (CrCl) < 30 mL/min or end-stage renal disease (ESRD), active malignancy, concomitant systemic antibiotic therapy, or immunocompromised status (HIV/AIDS, active chemotherapy, organ or bone marrow transplant, moderate to high-dose steroids (≥ 0.3 mg/kg/day prednisone), or immunosuppressive agents (i.e. mycophenolate, methotrexate, adalimumab, etc.).

LH is an integrated delivery network consisting of four acute care hospitals and two specialty hospitals with a total of 1423 beds in Fort Myers and Cape Coral, FL. Patients with a positive *C. difficile* result based on Xpert *C. difficile*/Epi (Cepheid, Sunnyvale, CA) were included in the study.

SMH is an 806-bed regional medical center, including a network of outpatient, long-term care, and rehabilitation centers, serving approximately 800,000 patients each year. CDAD was assessed at SMH using Xpert *C. difficile*/Epi *C. diff* Quik Chek Complete^®^ (TECHLAB, Blacksburg, VA) with reflex PCR using Xpert *C. difficile*/Epi (Cepheid, Sunnyvale, CA).

LH and SMH evaluated the impact of a treatment pathway targeting fidaxomicin for patients based on ATLAS Score and CDAD episode between 1/5/15 and 11/2/15 (Tables 1, 2). Patients were included if they were 18 years of age or older with laboratory confirmed CDAD admitted to the hospital, with a clinical diagnosis consistent with CDAD, seen by infectious diseases (ID) service and received at least 3 days of fidaxomicin. Patients with fulminant disease, ileus, toxic megacolon, receipt of alternative CDAD therapy such as tigecycline or IVIG, greater than 24 h of metronidazole or vancomycin or had missing ATLAS score criteria were excluded.

**Table 1:** ATLAS scoring system (Florida)

Parameter	0 points	1 point	2 points
Age (years)	< 60	60–79	≥ 80
Treatment with systemic antibiotics for ≥ 1 day upon CDAD diagnosis or 48 h prior	No	–	Yes
Leukocytosis	< 16,000	16,000–25,000	> 25,000
Albumin (g/dL)	> 3.5	2.6–3.5	≤ 2.5
Serum creatinine (mg/dL)	≤ 1.3	1.4–2	> 2

**Table 2:** *Clostridium*-*difficile* associated diarrhea treatment pathway using ATLAS scoring (Florida)

ATLAS score	ATLAS classification	First episode	First recurrence	≥ 2 recurrences
0	Mild CDAD	Metronidazole 500 mg PO/IV q8h	Vancomycin 125–250 mg PO q6h	Fidaxomicin 200 mg PO BID or Vancomycin taper^b^
1				
2				
3	Moderate CDAD	Vancomycin 125–250 mg PO q6h	Fidaxomicin 200 mg PO BID	Fidaxomicin 200 mg PO BID or Vancomycin taper^b^
4				
5	Severe CDAD	Fidaxomicin 200 mg PO BID	Fidaxomicin 200 mg PO BID	Fidaxomicin 200 mg PO BID or Vancomycin taper^b^
6				
7				
8				
9				
10				
Any	Fulminant CDAD^a^	Fidaxomicin 200 mg PO BID + Metronidazole 500 mg IV q8h	Fidaxomicin 200 mg PO BID + Metronidazole 500 mg IV q8h	(Fidaxomicin 200 mg PO BID or Vancomycin taper^b^) + Metronidazole 500 mg IV q8h

^a^CDAD in presence of hypotension/shock (SBP < 90 mmHg requiring vasopressor therapy), ileus, or toxic megacolon

^b^Vancomycin taper: vancomycin 125 mg PO q6h × 10 days, followed by vancomycin 125 mg PO q12h × 7 days, followed by vancomycin 125 mg PO q24h × 7 days, then vancomycin 125 mg PO every 3 days × 14 days

Patients in all centers were assessed to determine adherence to pathway protocol, recurrence at 30 and 60 days, readmission and clinical cure. Recurrence was defined as a new clinical diagnosis of CDAD or receipt of new CDAD therapy after completion of initial course after 14 days from start but within 30 and 60 days post treatment cessation. Clinical cure was based on assessment of one or more of the following criteria: resolution of diarrhea (less than or equal to three unformed stools for two consecutive days) maintained throughout the treatment course and 2 days afterward or until hospital discharge, documentation of clinical resolution in the medical record, no requirement for additional CDAD therapy (including the need for a switch in CDAD therapy) on or before Day 14.

Descriptive statistics were used to describe the patient population and the outcomes among patients adhering to the treatment pathway. Continuous variables were reported as means and standard deviations. Categorical variables were reported as frequency and percent.

The institutional review board at each participating institution approved the study.

## Results

### WellStar

**Fig. 1:**
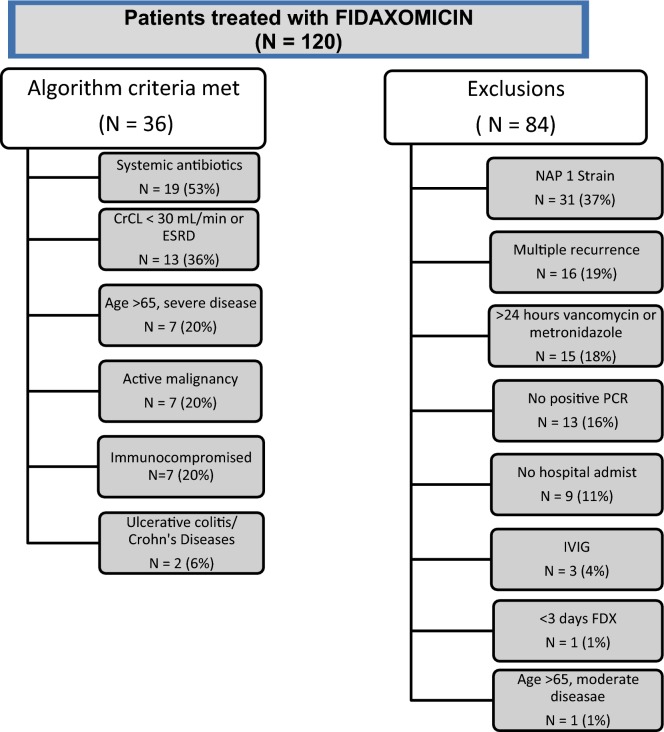
Inclusion and exclusions for WellStar fidaxomicin pathway

There were 120 patients who received fidaxomicin at a participating WellStar institution within the study timeframe, but only 36 (30%) met inclusion/exclusion criteria. Exclusions were due to NAP1 strain (37, 31%), multiple recurrences (24, 20%), prior CDAD treatment (22, 18%), and absent/missing PCR (19, 16%) (Fig. 1). Mean age was 68.6 years (16.7 SD, range 24–91). Half of the patients were male and the majority (61%) was Caucasian. Most of the patients were admitted from the community (75%) and a small percentage had a previous hospitalization (14%) or ICU stay (8%) within the past 30 days. The patients included in the analysis had several comorbidities including moderate to severe renal disease (56%), diabetes (53%) and cardiovascular disease (47%) (Table 3).

**Table 3:** Patient demographics and *Clostridium difficile*-infection related outcomes

	WellStar	Florida
Patient characteristics	N = 36	N = 28
Age	68.6 ± 16.7	82 ± 10.2
Gender: Male	18 (50%)	14 (50%)
Race		
Caucasian	22 (61%)	23 (82%)
African American	5 (14%)	1 (3.0%)
Other	9 (25%)	4 (14%)
Admission source		
Community	27 (75%)	15 (57%)
Other healthcare facility	9 (25%)	12 (43%)
Previous hospitalization	5 (14%)	17 (61%)
Episode		
Initial episode	32 (89%)	20 (71%)
First recurrence	4 (11%)	6 (21%)
> 2 recurrences	NA	2 (7%)
CDAD present within 24 h	15 (42%)	17 (61%)
Top comorbidities		
Moderate to severe renal disease	20 (56%)	6 (18%)
Diabetes	19 (53%)	9 (27%)
Cardiovascular disease	17 (47%)	15 (54%)
Fidaxomicin treatment (days)	8.4 ± 3.6	7.9 ± 5.2
Outcomes		
Pathway adherence	36/120 (30%)	28/142 (20%)
Recurrence 30 day	0	2 (7%)
Recurrence 60 day	0	3 (9.7%)
Readmission	5 (14%)	6 (21%)
Readmission related to CDAD	0	3 (50%)
Clinical cure	30 (83%)	26 (93%)

This was the first CDAD episode for 32 (89%) of patients and the CDAD was present within 24 h of admission for 15 (42%) of the patients. Patients received an average of 8.4 ± 3.6 days of fidaxomicin. Most patients (83%) were considered clinical cure based on resolution of diarrhea (37%), clinical documentation (23%) and/or no additional CDAD treatment (50%). There were no recurrences at 30 or 60 days and there were five readmissions, but none were related to CDAD.

### Florida

There were 142 patients who received fidaxomicin during the study period, but only 28 (20%) met study criteria. Patients were excluded due to receiving greater than 24 h of vancomycin or metronidazole (57, 40%), not meeting ATLAS pathway criteria (31, 22%), receiving less than 3 days of fidaxomicin (11, 8%), no ID consult (6, 4%), or having fulminant disease (6, 4%). Of the eligible patient population, the average age was 82 years (10.2 SD, range 57–100). Half were male and the majority was Caucasian (23, 82%). Many were admitted from another care facility (12, 43%) and the majority had a previous hospitalization (17, 61%). The patients included had several comorbidities including cardiovascular disease (54%), diabetes (27%) and moderate to severe renal disease (18%). The majority (26, 93%) of patients had an ATLAS score ≥ 5.

This was the first episode for 20 (71%) and the majority (17, 61%) had CDAD within 24 h of admission. Patients received an average of 7.9 ± 5.2 days of fidaxomicin. Most patients (26, 93%) were considered to have a clinical cure, and two (6%) had recurrence at 30 days. Six (19%) patients were readmitted within 30 days, of whom three (50%) were related to CDAD. All three of these patients had at least one previous CDAD episode prior to the index event.

## Discussion

The results from these two pathways show positive outcomes for the use of fidaxomicin in patients at high risk for CDAD recurrence. This data supports the benefits of fidaxomicin against CDAD. Although these two pathways used different criteria to identify patients at high risk, most of the patients included were older and had multiple comorbidities. Most patients presented with an initial CDAD episode (89% in WellStar, 71% in Florida) and first CDAD recurrent episodes (11%, 21%) and resulted in similar known recurrence rates [7, 15]. After completion of treatment, 30-day recurrence rates in the Florida pathway (7%) were similar to recurrence rates of studies that compared fidaxomicin to vancomycin [8] whereas the WellStar pathway resulted in no recurrent episodes. Clinical cure rates remained high in both pathways, 83% in WellStar and 93% in Florida.

These results are consistent with that of other CDAD studies [7, 15]. *C. difficile*-infection has become more prevalent and is a leading cause of morbidity and mortality in hospitalized patients [1, 15–18]. Due to its increased prevalence, there are significant financial burdens associated with the management of CDAD. These financial burdens are attributed to increases in treatment failures, recurrent disease and emergence of a hypervirulent strain [17, 19, 20]. Recurrent CDAD causes significantly increased mortality when compared to primary CDAD and can range from 20% from an initial episode to 60% after multiple recurrences [15, 16]. In order to decrease the burden of CDAD, alterations in current standards of treatment are necessary.

A limitation to these studies was poor adherence or exclusions to the CDAD treatment pathways (30%, 20%), which resulted in a smaller than expected sample size. In addition, these are retrospective studies and data is limited to what is collected in the normal course of care and entered into the patient’s clinical record. Nonetheless, these pathways allowed for evaluation of fidaxomicin use and demonstrate the difficulties of adherence and evaluation of institution-specific pathways across multiple institutions. CDAD scoring tools can aid in identifying severity of disease and allow for modification of treatment regimens to optimize patient outcomes.
